# Caspase-1 Inhibition Impacts the Formation of Chondrogenic Nodules, and the Expression of Markers Related to Osteogenic Differentiation and Lipid Metabolism

**DOI:** 10.3390/ijms22179576

**Published:** 2021-09-03

**Authors:** Alice Ramesova, Barbora Vesela, Eva Svandova, Herve Lesot, Eva Matalova

**Affiliations:** 1 Department of Physiology, University of Veterinary Sciences Brno, 612 42 Brno, Czech Republic; alice.ramesova@seznam.cz (A.R.); evabsvandova@gmail.com (E.S.); matalova@iach.cz (E.M.); 2 Laboratory of Odontogenesis and Osteogenesis, Institute of Animal Physiology and Genetics, Academy of Sciences, 602 00 Brno, Czech Republic; herve.lesot@gmail.com

**Keywords:** caspase-1, Cd36, osteoarthritis, cartilage, chondrocytes, inhibition, lipid metabolism

## Abstract

Caspase-1, as the main pro-inflammatory cysteine protease, was investigated mostly with respect to inflammation-related processes. Interestingly, caspase-1 was identified as being involved in lipid metabolism, which is extremely important for the proper differentiation of chondrocytes. Based on a screening investigation, general caspase inhibition impacts the expression of *Cd36* in chondrocytes, the fatty acid translocase with a significant impact on lipid metabolism. However, the engagement of individual caspases in the effect has not yet been identified. Therefore, the hypothesis that caspase-1 might be a candidate here appears challenging. The primary aim of this study thus was to find out whether the inhibition of caspase-1 activity would affect *Cd36* expression in a chondrogenic micromass model. The expression of *Pparg*, a regulator *Cd36*, was examined as well. In the caspase-1 inhibited samples, both molecules were significantly downregulated. Notably, in the treated group, the formation of the chondrogenic nodules was apparently disrupted, and the subcellular deposition of lipids and polysaccharides showed an abnormal pattern. To further investigate this observation, the samples were subjected to an osteogenic PCR array containing selected markers related to cartilage/bone cell differentiation. Among affected molecules, *Bmp7* and *Gdf10* showed a significantly increased expression, while *Itgam*, *Mmp9*, *Vdr*, and *Rankl* decreased. Notably, Rankl is a key marker in bone remodeling/homeostasis and thus is a target in several treatment strategies, including a variety of fatty acids, and is balanced by its decoy receptor Opg (osteoprotegerin). To evaluate the effect of Cd36 downregulation on *Rankl* and *Opg*, *Cd36* silencing was performed using micromass cultures. After *Cd36* silencing, the expression of *Rankl* was downregulated and *Opg* upregulated, which was an inverse effect to caspase-1 inhibition (and *Cd36* upregulation). These results demonstrate new functions of caspase-1 in chondrocyte differentiation and lipid metabolism-related pathways. The effect on the Rankl/Opg ratio, critical for bone maintenance and pathology, including osteoarthritis, is particularly important here as well.

## 1. Introduction

Chondrogenesis is a process of cartilage formation which plays a crucial role also in proper long bone development and elongation. Chondroblasts and chondrocytes surrounded by calcifying extracellular matrix participate at the cellular level [1]. The metabolism of lipids was shown to play an important role in the differentiation, maintenance, and health of cartilage [2]. Abnormal lipid deposition is shown to accompany several diseases including osteoarthritis (OA), the most prevalent chronic joint disorder associated with chondrocytes [3]. 

Cd36, the fatty acid translocase typical for hypertrophic chondrocytes, is an important molecule in lipid pathways [4]. Recently, alterations in *Cd36* expression in chondrocytes were achieved after general caspase inhibition [5,6]. Caspase-1, the major pro-inflammatory cysteine protease, appears as a good candidate to mediate this effect. 

Caspases, molecules traditionally associated with apoptosis and inflammation [7], are known from physiological chondrogenesis [6,8,9] as well as related pathologies [10]. The inflammatory caspase-1, originally named interleukin-1β converting enzyme, contains a caspase-recruitment domain (CARD), which enables its dimerization and thus activation [7]. Activated caspase-1 then acts as a protease, responsible for the activation of immature precursors of inflammatory cytokines, especially interleukin-1β [11]. Caspase-1 displays also other functions, including those involved in lipid and glucose metabolism [12]. The activation of caspase-1 occurs in pyroptosis, a rapid, caspase-1-dependent form of cell death. Pyroptosis is frequently induced by infected macrophages [11,13] and participates in osteoarthritis progression [10,14]. The link between lipid metabolism and caspase-1 is also supported by the fact that caspase-1-deficient mice develop obesity [15]. As such, caspase-1 is favored as a potential target molecule in clinical treatments [16]. 

Caspase-1 can be involved in both lipid metabolism and chondrocyte regulation, but the complexity of these processes is not yet clear. The present research was designed to contribute to a better understanding of the non-traditional engagement of caspase-1. First, the hypothesis that caspase-1 inhibition might modify *Cd36* expression was tested. Then, the impact of caspase-1 inhibition on differentiation pathways of chondrocytes was identified. Finally, silencing of *Cd36* siRNA was performed to investigate the effect of caspase-1 inhibition and *Cd36* downregulation on the expression of *Rankl* and *Opg* as critical molecules involved in osteogenic pathways.

## 2. Results

### 2.1. Caspase-1 Is Present in Developing Growth Plate

**Figure 1:**
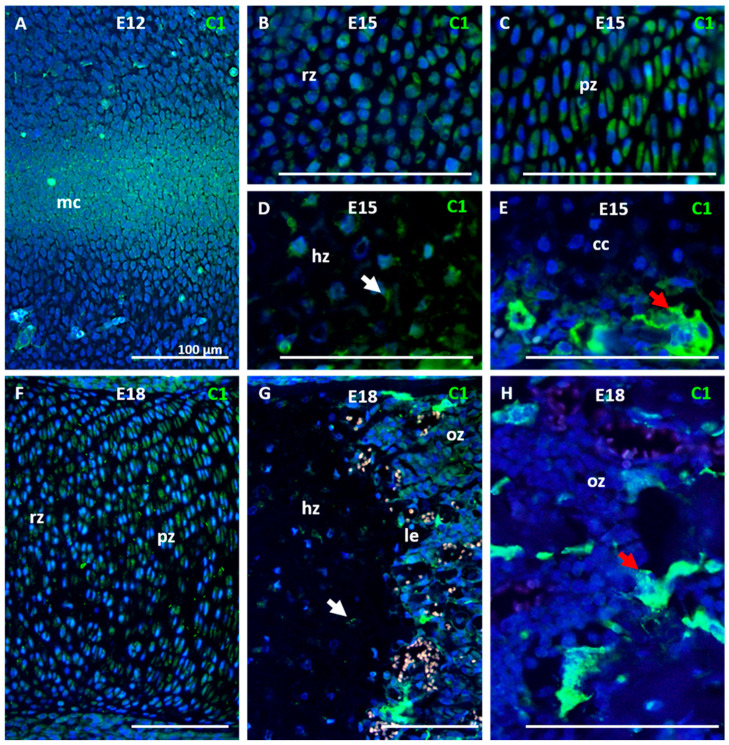
A temporospatial pattern of caspase-1 activation in the developing growth plate of a mouse forelimb. Detection of caspase-1 in condensed mesenchymal cells at the stage E12 (**A**). Detection of caspase-1 in resting (**B**), proliferating (**C**), and hypertrophic (**D**) zones of the growth plate at the stage E15. Detail of activation of caspase-1 in chondroclasts/osteoclasts penetrating to the primary ossification center (**E**) at the stage E15. Detection of caspase-1 in resting and proliferating (**F**), hypertrophic, and ossification (**G**) zones of the growth plate at the stage E18. Caspase-1 activation in osteoclasts of ossification zone at the stage E18 (**H**). The positive fluorescent signal is green, nuclei are counterstained by DAPI and are blue. The white arrows point to positive hypertrophic chondrocytes. The red arrows point to positive chondroclasts/osteoclasts. mc (mesenchymal condensation), rz (resting zone of the growth plate), pz (proliferating zone), hz (hypertrophic zone), cc (calcifying cartilage), oz (ossification zone). Scale bar = 100 μm.

To localize the activated caspase-1 during cartilage development, embryonic stages of mouse forelimbs E12, E15, and E18 were examined. At E12, caspase-1 was detected in the condensed mesenchyme of the future growth plate (Figure 1A). At the stage E15, caspase-1 was observed in resting (Figure 1B), proliferating (Figure 1C), and hypertrophic (Figure 1D) chondrocytes of the developing growth plate. Caspase-1 was not observed in the zone of calcifying cartilage but was activated in chondroclasts/osteoclasts penetrating the primary ossification center (Figure 1E). At the stage E18, when all zones (resting, proliferating, hypertrophic, and ossification) of the growth plate are present, caspase-1 was still activated in resting and proliferating chondrocytes (Figure 1F), but only a mild signal was observed in hypertrophic chondrocytes (Figure 1G). In the case of the ossification zone, caspase-1 was found predominantly in osteoclasts (Figure 1H). 

### 2.2. Caspase-1 Inhibition Affects the Structure of Forming Nodules in the Micromass Cultures

**Figure 2:**
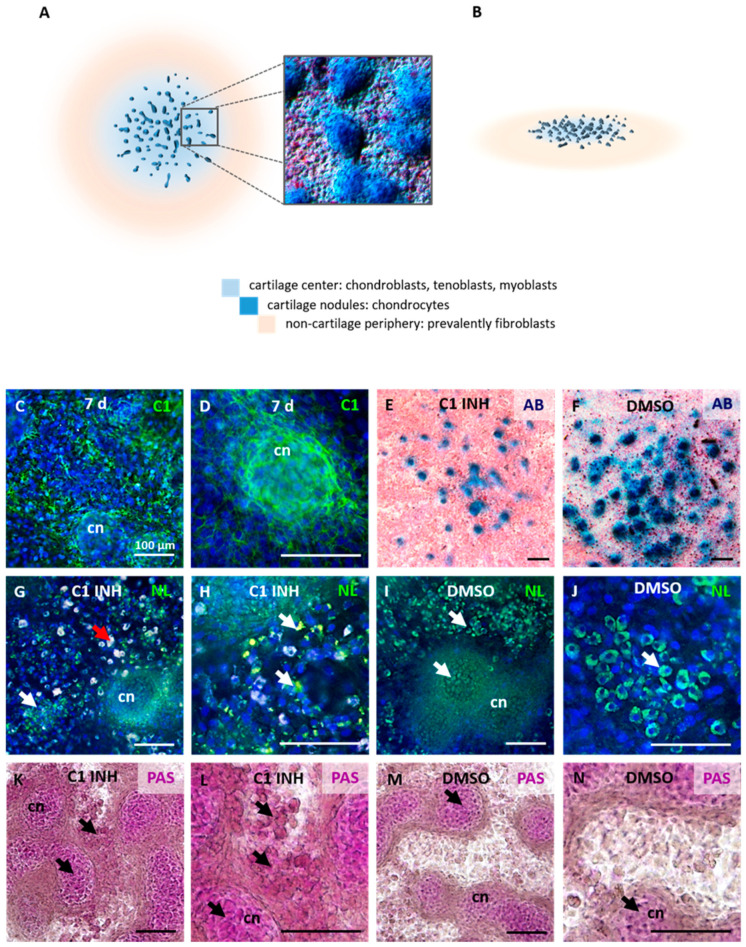
Characterization of micromass cultures treated by caspase-1 inhibitor. Scheme of micromass cultures (**A**,**B**), showing the arrangement of chondrogenic and non-chondrogenic cells in the culture. Activation of caspase-1 in the micromass cultures (**C**), predominantly in the highly differentiated central part containing chondrogenic nodules (**D**). Alcian blue (cartilage extracellular matrix)/eosin staining of caspase-1 inhibited (**E**) and control (**F**) micromass cultures. Lipid accumulation in cultures with inhibited caspase-1 (**G**,**H**) and control cultures (**I**,**J**). PAS staining was performed on caspase-1 treated (**K**,**L**) and control samples (**M**,**N**). The white arrows point to lipid accumulation, the red arrow to polysaccharide accumulation, and the black arrows to positive PAS staining. cn (cartilage nodule), AB (alcian blue), NL (neutral lipid stain), PAS (Periodic acid–Schiff). Scale bar = 100 μm.

Micromass culture is a primary cell culture providing a 3D microenvironment for chondrogenic studies [17]. For this purpose, cells obtained from embryonic tissues (E12 front limb) were seeded as high-density spots to develop chondrogenic nodules (Figure 2A,B).

The localization of active caspase-1 by immunofluorescence demonstrated its presence in micromass cultures (Figure 2C), predominantly in the central, highly differentiated part of the nodules (Figure 2D). In experimental conditions where caspase-1 was inhibited, a lower number of nodules with reduced size was observed (Figure 2E,F). This pointed to a decreased cell differentiation within the spots.

Additionally, lipid distribution in cultures with inhibited caspase-1 was confirmed by the detection of neutral lipids (Figure 2G–J). The samples treated by caspase-1 inhibitor showed decreased lipid staining and revealed an obvious auto-fluorescent signal attributed to polysaccharide accumulation (Figure 2G). To confirm this, PAS staining was used. In both groups, PAS staining was positive in cartilaginous nodules (Figure 2K–N). Moreover, the cultures with inhibited caspase-1 displayed increased PAS staining in the regions surrounding nodules (Figure 2L).

### 2.3. Inhibition of Caspase-1 Affects the Expression of Cd36 and Pparg Genes Connected with Lipid Metabolism

**Figure 3:**
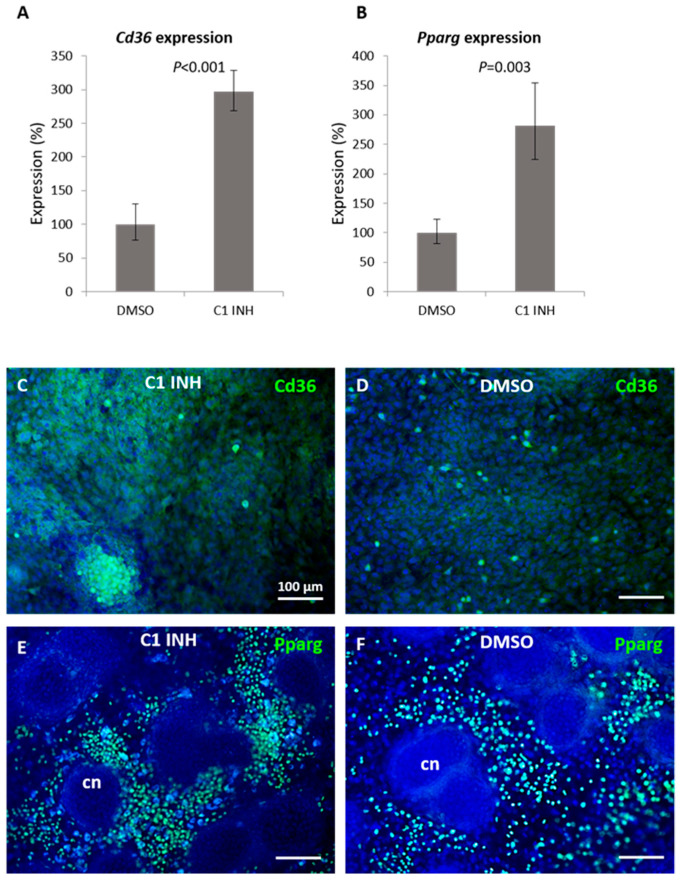
RNA expression of *Cd36* and *Pparg* and respective protein detection after caspase-1 inhibition in micromass cultures. Expression of *Cd36* (**A**) and *Pparg* (**B**) in micromass cultures after 6 days of cultivation with caspase-1 inhibitor. Immunofluorescent detection of Cd36 and Pparg in micromass cultures after 6 days of cultivation with caspase-1 inhibitor (**C**,**E**) and in control (DMSO) group (**D**,**F**). The positive fluorescent signal is green, nuclei counterstained by DAPI are blue. cn (cartilage nodule). Scale bar = 100 μm.

To test whether caspase-1 can be the key cysteine protease modulating the expression of *Cd36*, a selective caspase-1 inhibitor was applied. For these experiments, samples from the same litter were used to take into account the effects of maternal lipid metabolism on fetal growth [18]. Expression levels of *Cd36* were evaluated by qPCR along with *Pparg*, the master regulator of fatty acid storage and lipid/glucose metabolism. When cultured for 7 days in conditions where caspase-1 was inhibited, the expression of *Cd36* increased up to 300% compared to control (Figure 3A). Similarly, the expression of *Pparg* increased up to 280% after caspase-1 inhibition (Figure 3B). Cd36 protein was expressed in both groups although the staining was weaker in controls (Figure 3C,D). Pparg was detected in the nuclei of cells surrounding nodules, also with a weaker signal in controls (Figure 3E,F). Notably, the localization of Pparg-positive cells overlapped with areas of lipid accumulation. 

### 2.4. Caspase-1 Inhibition Alters the Expression of Osteogenic Genes

**Figure 4:**
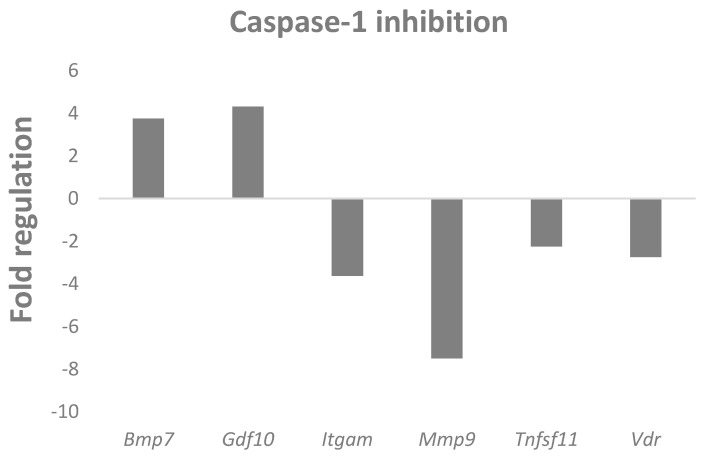
PCR array analysis of osteogenic gene expression in micromass cultures after 6 days of caspase-1 inhibition compared to control. Only osteogenic genes with statistically significant (*p* ≤ 0.05) changes in gene expression are showed.

Along with the participation of caspase-1 in lipid metabolism, the impact of caspase-1 inhibition on osteogenic expression was investigated. A pre-designed panel of osteogenesis-related genes, including many factors essential also for chondrogenesis, was used. The chondrogenic micromasses cultured in conditions where caspase-1 was inhibited were compared to controls. The PCR array analysis revealed statistically significant changes in the expression of six genes (Figure 4). The upregulated genes were *Gdf10* (Growth differentiation factor 10, fold regulation: 4.31, *p* = 0.019) and *Bmp7* (Bone morphogenetic protein 7, fold regulation: 3.75, *p* = 0.029). Downregulated genes included *Mmp9* (Matrix metallopeptidase 9, fold regulation: –7.5, *p* = 0.046), *Itgam* (Integrin alpha M, fold regulation: –3.62, *p* = 0.003), *Vdr* (Vitamin D receptor, fold regulation: –2.74, *p* = 0.031), and *Tnfsf11*, gene for Rankl (fold regulation: –2.24, *p* = 0.048). 

### 2.5. Caspase-1 Inhibition and Cd36 Silencing Impact the Expression of Rankl and Opg in an Opposing Trend

**Figure 5:**
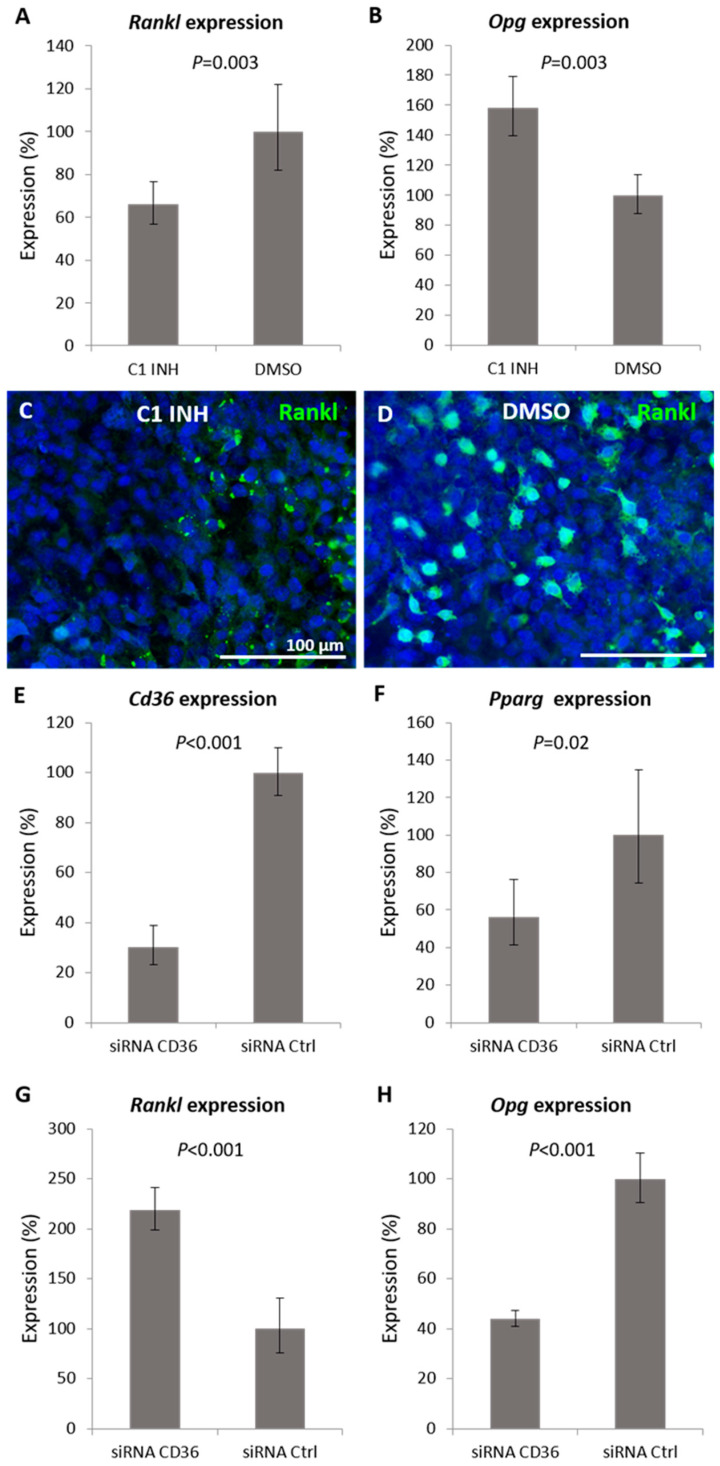
Expression of *Rankl* and *Opg* in caspase-1 inhibited or *Cd36* silenced micromass cultures. Gene expression of *Rankl* (**A**) and *Opg* (**B**) after 6 days of caspase-1 inhibition. Immunofluorescent detection of Rankl (green) in micromass cultures after 6 days of cultivation with caspase-1 inhibitor (**C**) and in control micromasses (**D**). Expression of *Cd36* (**E**), *Pparg* (**F**), *Rankl* (**G**), and *Opg* (**H**) after 6 days of *Cd36* silencing in micromass cultures. Scale bar = 100 μm.

Since *Rankl* was among the affected genes and the Rankl–Rank–Opg pathway is critical in cartilage and bone homeostasis, the expression of *Rankl* and *Opg* was further investigated. To specify the role of Cd36 in Rankl–Opg regulation, *Cd36* RNA silencing was performed in simultaneously running cultures. After caspase-1 inhibition (causing *Cd36* upregulation), *Rankl* expression decreased and *Opg* expression increased (Figure 5A,B). The expression trend at the protein level was confirmed for Rankl by immunofluorescence, showing a weak signal in caspase-1 inhibited groups (Figure 5C) while the control samples displayed positive cells (Figure 5D). *Cd36* silencing decreased the expression of *Cd36* to the level of 30% (*p* < 0.001) in treated cultures (Figure 5E). As a consequence, the expression of *Pparg* decreased to 60% (*p* = 0.02) compared to controls (Figure 5F). After *Cd36* silencing (with opposite effect to caspase-1 inhibition on *Cd36* expression), the expression of *Rankl* increased to 220% (*p* < 0.001) (Figure 5G), and *Opg* decreased to 45% (*p* < 0.001) (Figure 5H).

## 3. Discussion

Limb bud derived micromass cultures are the most common in vitro model to study chondrogenesis [17]. After one week in vitro, chondroblasts and differentiated chondrocytes were located in the central part of the micromass spot (3D nodules). The outer part of the spot as well as regions surrounding the nodules consisted of non-cartilaginous cells, primarily fibroblasts but also myoblasts and tenoblasts. Therefore, micromass cultures imitate the physiological cell interactions within tissues [19]. 

Cd36 is a membrane glycoprotein present on many cell types, including osteoblasts [20,21]. It is expressed also by hypertrophic chondrocytes [4]. Cd36 interferes with lipid metabolism as a multifunctional receptor for fatty acid uptake [22]. The modulation of Cd36 expression using the micromass model after general caspase inhibition has been reported earlier [5,6]. Until now, the effect of individual caspases on *Cd36* expression in cartilage has not been identified. In the present investigation, caspase-1 was a hot candidate because of its engagement in lipid metabolism [12,23] and abundant caspase-1 activation, which we identified during the formation of the growth plate. Additionally, caspase-1 is associated with arthritic diseases [10,24], where both inflammation and metabolic dysbalance play a role [25]. Inflammasome, in which caspase-1 is activated, was observed in many arthritic disorders involving the production of pro-inflammatory cytokines such as Il-1β [26]. Notably, the modulation of Cd36-Pparg pathways provides attractive options for the treatment of metabolic diseases [27], where caspase-1 is also a potential target molecule [16].

The upregulation of *Cd36* in samples where caspase-1 was inhibited confirmed the hypothesis that caspase-1 might be able to modulate *Cd36* expression. In addition, there was a remarkable alteration in the size and number of chondrogenic nodules compared to controls. Caspase-1 inhibition also caused an abnormal accumulation of lipid droplets in the cartilage nodules and cells surrounding the nodules. The caspase-1 associated pathways in lipid metabolism include, along with interleukins, SREBS (and lipid biosynthesis), FABS (and lipid absorption and VLDL secretion), SIRT1 (and adipogenesis and insulin resistance), and others [28]. Caspase-1 regulation of glucose and lipid metabolism acts through the cleavage of glycolytic enzymes and by direct or cytokine-dependent activation of transcriptional factors [12,29].

Furthermore, the regions surrounding the nodules showed a higher incidence of PAS-positive cells indicating an increased level of polysaccharides. This might be related to the observation [12] of a low level of triacylglyceride in the circulation of caspase-1 deficient mice compared to the wild type. Analysis of Cd36 and Pparg within the micromass cultures indicated a stronger signal in chondroblasts surrounding differentiated chondrocytes in the center of the nodules. In the same region, polysaccharide droplets were detected. Taken together, our results imply that caspase-1 in chondrocytes participates in the regulation of both lipid and glucose metabolism. 

To follow other functions of caspase-1 in chondrocytes, a PCR array of pre-selected markers was screened. There is not much information about the specific roles of inflammatory caspases in cell differentiation. The activation of caspase-1, so far, has been investigated in cases of myoblast and neuronal differentiation [30,31]. In caspase-1 inhibited micromass cultures, the most prominent decrease was detected in the expression of *Mmp9*. Although at a lower level, the same trend was observed in the expression of *Vdr*, which is associated with the expression of Mmps [32]. Moreover, both molecules can play a role in lipid metabolism [33,34]. *Itgam*, another downregulated factor after caspase-1 inhibition, participates in signaling causing reduced chondrocyte mineralization [35]. This could contribute to alterations in the phenotype of the inhibited micromasses. 

Notably, all the downregulated genes contribute to the progression of osteoarthritis. Mmp9 is connected with cartilage degradation and pathologies [36,37]. Vdr polymorphisms were associated with susceptibility for primary osteoarthritis [38]. Itgam affects the severity of osteoarthritis [35]. The effect of caspase-1 inhibition on cartilage maintenance involves also the most upregulated genes, *Bmp7* and *Gdf10*. Members of the Gdf family have been detected during cartilage formation and Gdf10 was suggested to play a role in mesenchymal condensation and chondrocyte maturation [39]. Gdf10 was also reported to attenuate the activity of Pparg [40]. Bmp7, in turn, prevents the progression of cartilage damage in a rabbit model of osteoarthritis [41]. From this point of view, caspase-1 inhibition would have a protective effect related to the progression and severity of osteoarthritis. 

Caspase-1 inhibition also decreased the Rankl/Opg ratio. The Rank-Rankl-Opg is a sophisticated regulatory system for bone remodeling [42]. Osteoblastic Rankl binds to its Rank receptor expressed by osteoclastic precursors and allows for their maturation, functional differentiation, and thus bone resorption. Opg is the decoy receptor, which interferes with the Rankl–Rank interaction and favors bone apposition. Therefore, the Rankl/Opg ratio is extremely important. 

Since Rankl and Opg are produced also by chondrocytes [43], these molecules are potential therapeutic targets, not only within anti-osteoporotic strategies, but also in the case of anti-osteoarthritis prediction, progression, and treatment [44,45]. In caspase-1 inhibited samples, *Rankl* was decreased and *Opg* was increased. *Cd36* silencing determined that the effect could be mediated via Cd36 (upregulated in the caspase-1 treated cultures), 

The present results demonstrate the activation of caspase-1 in chondrocytes in vivo as well as in in vitro systems. In the latter case, the inhibition of caspase-1 resulted in a significantly increased expression of *Cd36,* which, consequently, impacted the Rankl/Opg ratio in chondrogenic cultures. These findings allow for a better understanding of the protective role of caspase-1 inhibition regarding OA phenotypes [46]. 

The role of Cd36 in chondrocyte metabolism is not completely clear. So far, in bone, Cd36 was demonstrated as essential for proper osteoblast function since it affected the expression of some osteogenic genes such as Runx2, osterix, or osteocalcin [47]. In the cartilage, *Cd36* is expressed by chondrocytes and can be associated with cartilage repair in response to inflammatory stimuli [4]. The interplay of Cd36, Pparg, and Opg can be mediated through the Erk pathway, as observed in liver cells [48] but not yet elucidated in the cartilage.

Despite the mechanism depends on the cell type, the engagement of caspase-1 in cell differentiation crystallizes. This applies to L8 cells and myeloblasts, PC12 cells and neurons, as well as adipocytes, where the differentiation process is conveyed by IL-1β signaling [28]. Recently, caspase-1 was demonstrated to promote monocyte-macrophage differentiation by repressing PPARγ [49]. This is in agreement with our observation in chondrocytes pointing to increased *Pparg* expression after caspase-1 inhibition. Caspase-1 regulations related to macrophages and lipid metabolism are another link between chondrocyte maintenance and disorders such as in the case of osteoarthritis [50]. 

Taken together, the results presented here suggest caspase-1 to be a molecule with a major impact on the regulation of cartilage development and maintenance, at least regarding inflammatory and metabolic functions. Modulations of caspase-1 activity thus open further applications including clinical trials [16]. 

## 4. Materials and Methods

### 4.1. Micromass Cultures

Cells for micromass cultures were obtained from mouse forelimbs at embryonic day (E) 12. Fresh post mortem limbs were removed, cut in pieces, and incubated for 1–2 h at 37 °C with Dispase (Gibco, New York, NY, USA, final activity 1 U/mL). Cells, at a concentration of 2 × 10^7^/mL, were spotted in 10 µL drops on the culture plate. The culture medium, which supports chondrogenic differentiation, was composed of DMEM (Sigma-Aldrich, Waltham, MA, USA) and Nutrient Mixture F12 (Sigma-Aldrich) in a ratio 2:3, 10% FBS (Sigma Aldrich), Penicillin/streptomycin (Sigma-Aldrich, final concentration 100 U/mL and 100 µg/mL), L-Glutamine (Sigma-Aldrich, final concentration 2 mM), β-Glycerol phosphate (Sigma-Aldrich, final concentration 10 mM), and Ascorbic acid (Sigma-Aldrich, final concentration 50 µg/mL). Cells were cultured without treatment overnight to adhere to the surface. For caspase-1 inhibition, micromasses were cultured in the presence of pharmacological inhibitor Z-WEHD-FMK (FMK002, R&D Systems, Minneapolis, MN, USA) at a concentration of 100 μM, according to the manufacturer’s recommendation and previous studies [9]. In the controls, DMSO, the inhibitor vehicle, was added. The culture medium with caspase inhibitor or DMSO was changed every second day during 6 days of culture. The experiments were performed in four biological replicates. 

### 4.2. SiRNA Gene Silencing

Micromass cultures were prepared as described above. The next day, cells were transfected with 20 nM Stealth siRNAs set of 3 Cd36 (IDs: MSS202775, MSS202776, MSS202777 Catalog No. 1320001, Ambion, Austin, TX, USA) using Lipofectamine RNAiMAX Reagent (13778, Life Technologies, Carlsbad, CA, USA) according to the producer´s instructions. Negative Control No. 1 siRNA (Catalog No. 4390843, Ambion) was used as a control. Cells were treated for 6 days; the medium with siRNA-Lipofectamine complexes was changed every 48 h of culture.

### 4.3. RNA Isolation, PCR Array, Real-Time PCR

The cultured cells were harvested into 350 µL RLT lysis buffer (Qiagen, Hilden, Germany) with β-mercaptoethanol (Sigma-Aldrich). RNA was isolated by RNeasy Kit (Qiagen), mRNA was transcribed into cDNA using SuperScript VILO (Invitrogen, Waltham, MA, USA). The Osteogenesis PCR Array (PAMM-026Z, Qiagen) was used for the analysis of gene expression after caspase-1 inhibition or after *Cd36* silencing in micromass cultures. The panel of housekeeping genes included: *Actb*, *B2m*, *Gapdh, Gusb*, and *Hsp90ab1*. The PCR Array format also included positive and negative controls.

Real-time PCR was performed in 10 μL of a final reaction mixture containing the one-step GB Ideal PCR Master Mix (Generi Biotech, Hradec Kralove, Czech Republic). Cd36 (Mouse *Cd36*, Mm00432403_m1), PPARγ (Mouse *Pparg*, Mm00440940_m1), Rankl (Mouse *Tnfsf11*, Mm00441906_m1), and Opg (Mouse *Tnfrsf11b*, Mm00435454_m1) expression was detected by using a TaqMan Gene Expression Assay (Thermo Fisher Scientific, Waltham, MA, USA) with normalization based on actin levels (Mouse *Actb*, Mm02619580_g1).

### 4.4. Immunofluorescence and Immunocytofluorescence

Mouse front limbs (CD1 strain) were collected as fresh post mortem samples. Stages E12, E15, and E18 were examined. Histological sections were deparaffinized in xylene and rehydrated in a gradient series of ethanol. Sections were pre-treated in citrate buffer (10 min/98 °C) for antigen retrieval and then incubated with Caspase-1 p20 (Cleaved Asp296) Antibody (PA5-99390, Thermo Fisher Scientific) overnight. The primary antibody was followed by incubation with secondary anti-rabbit antibody Alexa Fluor^®^ 488 (Thermo Fischer Scientific, Waltham, MA, USA) for 40 min at RT. Nuclei were detected by ProLong^®^ Gold Antifade reagent with DAPI (Thermo Fischer Scientific).

For immunocytofluorescence, micromass cultures were grown on culture glass and fixed by 4% paraformaldehyde. Primary antibodies for Caspase-1 p20 (PA5-99390, Thermo Fisher Scientific), Cd36 (PA1-16813, Thermo Fisher Scientific), Pparg (2443, Cell Signaling Technology, Danvers, MA, USA), and Rankl (PA5-110268, Thermo Fisher Scientific) were diluted in the range 1:50–1:200 and were applied overnight/4 °C. Alexa Fluor^®^ 488 or 568 (A11034, A10037, Thermo Fischer Scientific) was diluted at 1:200 and then applied for 40 min/RT. The cytoskeleton was visualized by ActinGreenTM 488 ReadyProbesTM Reagent (Thermo Fischer Scientific), and nuclei were detected by ProLong^®^ Gold Antifade reagent with DAPI (Thermo Fischer Scientific).

### 4.5. Staining of Micromass Cultures

Micromass cultures grown on glass were fixed by 4% PFA. Alcian blue and eosin staining was used for visualization of micromass structure and differentiation. Neutral lipids were detected by HCS LipidTOX™ Green Neutral Lipid Stain (H34475, Thermo Fisher Scientific) diluted 1:500 in PBS buffer and observed under a fluorescent microscope. For detection of polysaccharides, Periodic Acid-Schiff (PAS) staining was used. Fixed micromass cultures were incubated with Schiff reagent for 5 min and then counterstained by Harris hematoxylin. 

### 4.6. Statistical Analysis

PCR Arrays data were statistically evaluated by Qiagen Gene Globe as recommended by the manufacturer (available online https://geneglobe.qiagen.com/us/analyze, accessed on 22 January 2021). Statistical significance was determined as *p* < 0.05, and the threshold of fold regulation was ±2. Three biological replicates were evaluated in each group. Real-time PCR expression levels were calculated using the ∆∆CT method and results were analyzed using a two-tailed *t*-test.

## Data Availability

Not applicable.
